# Somatic gene mutations involved in DNA damage response/Fanconi anemia signaling are tissue- and cell-type specific in human solid tumors

**DOI:** 10.3389/fmed.2024.1462810

**Published:** 2024-10-03

**Authors:** Sudhir Kumar Rai, Wei Du, Jun Zhang, Herbert Yu, Youping Deng, Peiwen Fei

**Affiliations:** ^1^University of Hawaii Cancer Center, University of Hawaii, Honolulu, HI, United States; ^2^John A. Burns School of Medicine, University of Hawaii, Honolulu, HI, United States; ^3^Division of Malignant Hematology and Medical Oncology, University of Pittsburgh School of Medicine, UPMC Hillma Cancer Center, Pittsburgh, PA, United States; ^4^Department of Pathology and Laboratory Medicine, Mayo Clinic, Arizona Campus, Phoenix, AZ, United States

**Keywords:** DNA Damage Response (DDR), Fanconi anemia (FA) signaling, genome instability, DNA interstrand cross-links, somatic mutation ATR, BLM, RAD18

## Abstract

With significant advancements in the study of DNA Damage Response (DDR) and Fanconi Anemia (FA) signaling, we previously introduced the term “FA signaling” to encompass “all signaling transductions involving one or more FA proteins.” This network has now evolved into the largest cellular defense network, integrating over 30 key players, including ATM, ATR, BLM, HRR6, RAD18, FANCA, FANCB, FANCC, BRCA2, FANCD2, FANCE, FANCF, FANCG, FANCI, BRIP1, FANCL, FANCM, PALB2, RAD51C, SLX4, ERCC4, RAD51, BRCA1, UBE2T, XRCC2, MAD2L2, RFWD3, FAAP20, FAAP24, FAAP100, and CENPX. This system responds to both endogenous and exogenous cellular insults. However, the mutational signatures associated with this defense mechanism in non-FA human cancers have not been extensively explored. In this study, we report that different types of human cancers are characterized by distinct somatically mutated genes related to DDR/FA signaling, each accompanied by a unique spectrum of potential driver mutations. For example, in pan-cancer samples, ATM emerges as the most frequently mutated gene (5%) among the 31 genes analyzed, with the highest number of potential driver mutations (1714), followed by BRCA2 (4% with 970 putative driver mutations). However, this pattern is not universal across specific cancer types. For example, FANCT is the most frequently mutated gene in breast (14%) and liver (4%) cancers. In addition, the alteration frequency of DDR/FA signaling due to these mutations exceeds 70% in a subtype of prostate cancer, with each subtype of brain, breast, lung, and prostate cancers displaying distinct patterns of gene alteration frequency. Furthermore, these gene alteration patterns significantly impact patient survival and disease-free periods. Collectively, our findings not only enhance our understanding of cancer development and progression but also have significant implications for cancer patient care and prognosis, particularly in the development of effective therapeutic strategies.

## Introduction

Deficiencies in the DNA Damage Response (DDR) pathway result in diverse unrepaired DNA lesions, leading to replication stress, increased genome instability, and ultimately, tumorigenesis (1). To maintain genome stability, eukaryotic cells have evolved a robust defense signaling network that coordinates multiple DNA repair mechanisms, such as the DDR/Fanconi anemia (FA) signaling pathway (2–9).

Numerous studies have shown that FA, an autosomal recessive genetic disorder, is a hallmark consequence of accumulated unrepaired DNA lesions, including interstrand cross-links and single- or double-strand breaks (3, 10). First reported in 1927 by Swiss pediatrician Guido Fanconi, FA is a rare genetic disease characterized by bone marrow failure, developmental abnormalities, and increased cancer susceptibility, with an occurrence rate of 1 in 100,000 people in the European population (11, 12).

Cells derived from FA patients exhibit distinct chromosomal abnormalities, such as triradial and/or quadriradial chromosomes, which are used for clinical diagnosis. These cells are also hypersensitive to DNA-damaging agents, particularly interstrand cross-linking agents such as mitomycin C (MMC) and diepoxybutane (DEB), which is pertinent to precision oncology by guiding oncologists in prescribing appropriate cancer treatments for patients with FA-relevant gene mutations (4, 13–16).

Advancements in DNA sequencing and genetic testing have highlighted the important roles of somatically mutated DDR/FA-relevant genes in cancer development and progression among patients with or without FA. Multiple studies have demonstrated new mutations in DDR/FA-related genes across various cohorts of cancer patients without FA (17–20).

An interesting cohort study involving 181 FA patients from Germany demonstrated an increased risk of cancers in the esophagus (*n* = 6,281), vulvar (*n* = 2,411), head and neck (*n* = 240), breast (*n* = 34), and brain (*n* = 23), particularly due to mutations in complementation groups G vs. A (relative hazard = 2.2) and C vs. A (relative hazard = 5.4) (21).

Despite these findings, the distribution of DDR/FA gene mutations in cancers among non-FA patients remains underexplored. Further investigation in this area could significantly enhance our understanding of the broader translational impact of these mutations.

This current report highlights the patterns of somatic mutations in DDR/FA-relevant genes across various human cancers, with a focus on both pan-cancer samples and specific tissue or cell types. By examining over 30 core and closely related DDR/FA genes—including ATM, ATR, BLM, HRR6, FANCA, FANCB, FANCC, BRCA2, FANCD2, FANCE, FANCF, FANCG, FANCI, BRIP1, FANCL, FANCM, PALB2, RAD51C, SLX4, ERCC4, RAD51, BRCA1, UBE2T, XRCC2, MAD2L2, RFWD3, FAAP20, FAAP24, FAAP100, and CENPX—we found their mutation rates and the consequential alterations in DDR/FA signaling frequency are tissue- and cell-type specific. These mutations are significantly associated with cancer patient prognosis. We believe our study will enhance the understanding of cancer etiology and treatment, ultimately contributing to the development of more effective strategies to care for cancer patients.

## Results

### ATM is the most frequently mutated gene among the 30-plus genes involved in DDR/FA signaling, followed by BRCA2/FANCD1 across pan-human cancers

Cancer is a unique genetic disease characterized by uncontrolled cell growth due to genetic mutations. These mutations can occur in various genes that regulate cell fates, ultimately leading to neoplastic transformation. Accumulated studies indicate that DDR/FA signaling is an important biological model system for studying the underlying mechanisms of neoplasm and identifying different therapeutic targets to improve therapeutic strategies.

This system involves over 30 gene products, including ATM, ATR, BLM, RAD6/HRR6/UBE2A, RAD18, FANCA, FANCB, FANCC, BRCA2/FANCD1, FANCD2, FANCE, FANCF, FANCG, FANCI, BRIP1/FANCJ, FANCL, FANCM, PALB2/FANCN, RAD51C/FANCO, SLX4/FANCP, ERCC4/FANCQ, RAD51/FANCR, BRCA1/FANCS, UBE2T/FANCT, XRCC2/FANCU, MAD2L2/FANCV, RFWD3/FANCW, FAAP20, FAAP24, FAAP100, and CENPX (2, 3, 22). However, the mutational status of these genes in human cancer has not been extensively studied.

**Figure 1:**
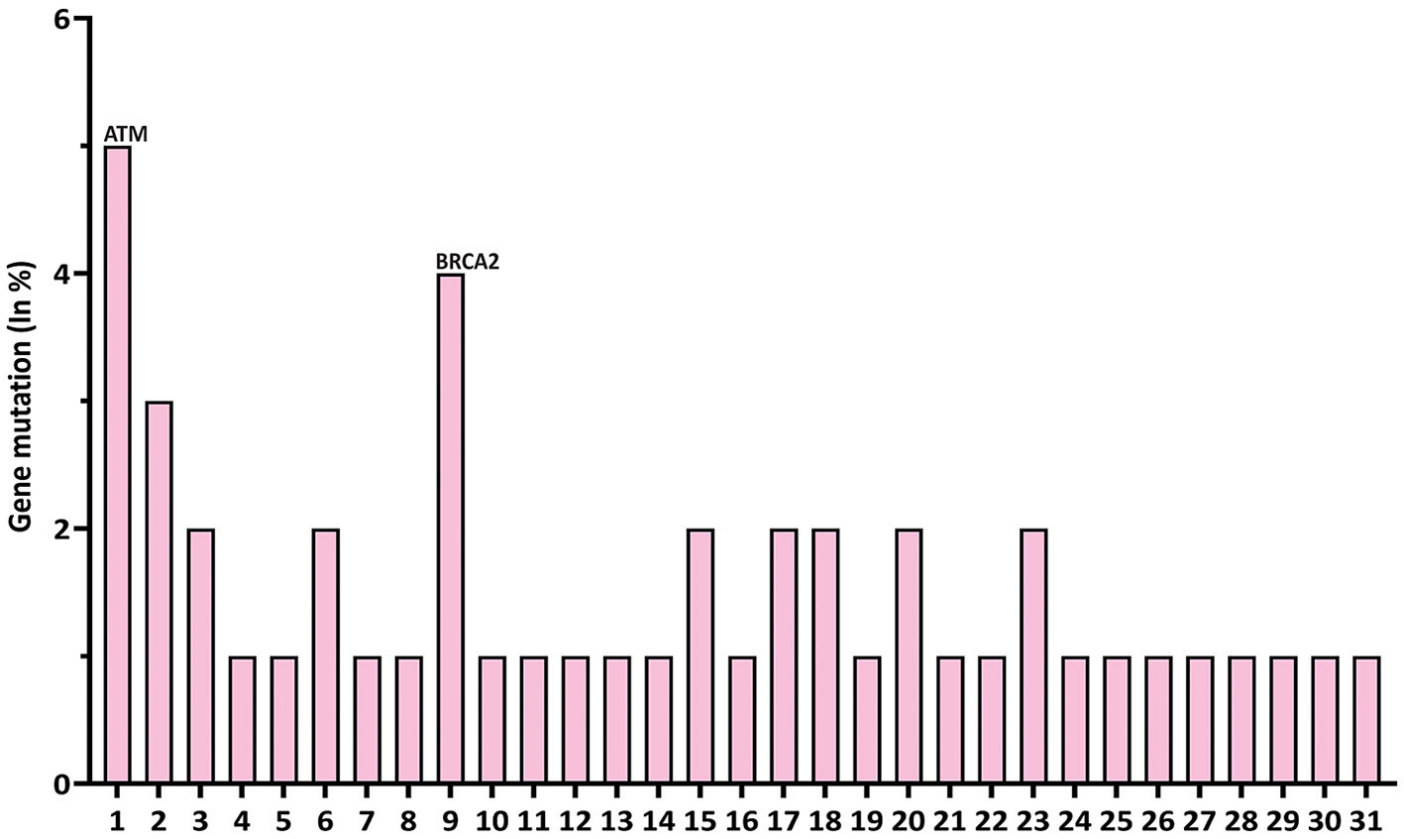
Gene mutation rates of 31 DDR/FA signaling-relevant genes in pan-human cancers. At the left of each gene name, the numbers 1–31 represent DDR/FA-relevant genes studied and indicated in the figure. The number indicated at the right of each gene name was the number of putative drive mutations found in this set of pan-human cancer samples studied. (1) ATM(**1714**), (2) ATR(456), (3) BLM(249), (4) UBE2A(0), (5) RAD18(0), (6) FANCA(221), (7) FANCB(0), (8) FANCC(82), (9) BRCA2(**970**), (10) FANCD2(41), (11) FANCE(25), (12) FANCF(9), (13) FANCG(19), (14) FANCI(0), (15) BRIP1(218), (16) FANCL(36), (17) FANCM(0), (18) PALB2(228), (19) RAD51C(62), (20) SLX4 (97), (21) ERCC4(79), (22) RAD51(31), (23) BRCA1(501), (24) UBE2T(0), (25) XRCC2(72), (26) MAD2L2(0), (27) RFWD3(0), (28) FAAP20(0), (29) FAAP24(0), (30) FAAP100(0), and (31) CENPX(0) (% could be equal or a bit less than the number indicated, rounded when >50%). The bold values indicate gene with high mutation rate.

We first analyzed DNA sequencing data from 76,639 pan-cancer samples available in public databases (23, 24). By querying 31 DDR/FA-relevant genes, we found that ATM had the highest mutation rate (5%), followed by BRCA2/FANCD1 (4%), with the remaining 29 genes showing lower mutation rates (2%, 1%, or less; Figure 1).

Somatic mutations in human cancer can be classified as either driver or passenger mutations. Driver mutations directly contribute to cancer initiation, progression, and maintenance by providing a selective growth advantage to cancer cells.

In contrast, passenger mutations are random genetic alterations incapable of providing a growth advantage.

To this end, we further investigated the nature of these genetic variations in the 31 genes studied. As shown in Table 1, ATM had 1,714 driver mutations, followed by 970 in BRCA2/FANCD1 (Table 1). These findings suggest that targeting ATM and/or BRCA2 may be a more cost-effective approach to developing new therapeutic strategies for treating human cancers in general.

**Table 1:** Rates of DDR/FA signaling relevant gene mutations in each type of human cancer.

**Cancer type**	**Breast**		**Lung**		**Bladder**		**Brain**		**Kidney**	
**31 genes**	***n*** = **11,657**	**Driver Mt No**.	***n*** = **14,261**	**Driver Mt No**.	***n*** = **5,276**	**Driver Mt No**.	***n*** = **8,205**	**Driver Mt No**.	***n*** = **3,611**	**Driver Mt No**.
ATM	4	103	**8**	469	**13**	264	2	45	2	40
ATR	4	45	6	96	7	45	2	29	1	9
BLM	4	22	2	21	4	18	1	14	< 1	1
UBE2A/HHA6	< 1	0	2	0	1	0	< 1	0	< 1	0
RAD18	3	0	1	0	7	0	< 1	0	**3**	0
FANCA	4	25	3	36	5	34	1	15	< 1	0
FANCB	1	0	2	0	2	0	1	0	< 1	0
FANCC	< 1	8	1	14	2	6	< 1	4	< 1	0
FANCD1/BRCA2	5	147	4	136	10	102	**3**	27	1	1
FANCD2	4	32	1	7	11	16	2	20	**3**	11
FANCE	2	1	1	9	2	5	< 1	4	< 1	18
FANCF	3	4	1	4	2	4	< 1	0	< 1	2
FANCG	2	2	3	4	3	4	< 1	1	< 1	0
FANCI	4	0	2	0	3	0	< 1	0	< 1	0
FANCJ/BRIP1	9	46	3	63	5	28	1	24	< 1	1
FANCL	2	4	2	5	3	5	< 1	3	< 1	1
FANCM	2	0	6	0	4	0	< 1	0	< 1	0
FANCN/PALB2	4	25	2	43	4	22	< 1	5	< 1	4
FANCO/RAD51C	7	26	1	30	3	22	< 1	11	< 1	0
FANCP/SLX4	6	13	3	28	6	19	< 1	2	< 1	12
FANCQ/ERCC4	4	10	2	24	3	17	< 1	5	< 1	3
FANCR/RAD51	2	2	< 1	8	2	2	< 1	0	< 1	0
FANCS/BRCA1	4	102	3	82	5	70	2	18	< 1	2
FANCT/UBE2T	**14**	0	3	0	1	0	1	0	< 1	0
FANCU/XRCC2	1	1	2	10	2	9	< 1	6	< 1	0
FANCV/MAD2L2	2	0	< 1	0	1	0	< 1	0	< 1	0
FANCW/RFWD3	2	0	2	0	3	0	< 1	0	< 1	0
FAAP20	3	0	1	0	2	0	< 1	0	< 1	0
FAAP24	2	0	4	0	3	0	< 1	0	< 1	0
FAAP100	4	0	3	0	3	0	1	0	< 1	0
CENPX	6	0	2	0	2	0	< 1	0	< 1	0
**Cancer type**	**Prostate**		**Eso/stomach**		**Bowel**		**Liver**		**Skin**	
**31 genes Mt% and driver**	***n*** = **10,998**	**Driver Mt No**.	***n*** = **4,994**	**Driver Mt No**.	***n*** = **7,661**	**Driver Mt No**.	***n*** = **1,829**	**Driver Mt No**.	***n*** = **3,279**	**Driver Mt No**.
ATM	5	226	**8**	200	**8**	369	**4**	23	10	93
ATR	2	40	6	84	4	127	3	10	10	58
BLM	< 1	9	4	63	3	73	1	2	6	17
UBE2A/HHA6	< 1	0	5	0	< 1	0	< 1	0	1	0
RAD18	2	0	3	0	2	0	< 1	0	2	0
FANCA	3	18	3	22	3	30	1	3	5	21
FANCB	2	0	**7**	0	2	0	2	0	3	0
FANCC	< 1	10	2	21	1	24	< 1	0	2	10
FANCD1/BRCA2	**6**	279	**8**	140	7	197	3	5	**11**	54
FANCD2	2	6	4	29	3	23	1	0	6	28
FANCE	1	4	2	20	1	10	2	1	4	6
FANCF	< 1	0	< 1	3	1	0	< 1	2	1	1
FANCG	2	0	3	6	2	9	< 1	1	3	8
FANCI	< 1	0	4	0	3	0	1	0	5	0
FANCJ/BRIP1	1	32	3	21	2	46	3	4	7	40
FANCL	1	1	1	4	1	3	< 1	0	1	3
FANCM	1	0	6	0	5	0	3	0	6	0
FANCN/PALB2	1	27	2	37	2	55	< 1	1	4	14
FANCO/RAD51C	< 1	0	1	3	1	7	2	3	3	8
FANCP/SLX4	2	9	4	27	5	24	2	1	8	15
FANCQ/ERCC4	< 1	9	2	29	2	24	1	4	3	14
FANCR/RAD51	< 1	1	< 1	1	< 1	13	< 1	1	2	3
FANCS/BRCA1	1	39	3	38	3	65	2	9	7	41
FANCT/UBE2T	2	0	2	0	< 1	0	**4**	0	3	0
FANCU/XRCC2	< 1	8	2	27	1	30	1	0	3	9
FANCV/MAD2L2	< 1	0	2	0	< 1	0	1	0	2	0
FANCW/RFWD3	3	0	1	0	2	0	< 1	0	2	0
FAAP20	2	0	2	0	< 1	0	2	0	2	0
FAAP24	< 1	0	4	0	1	0	< 1	0	< 1	0
FAAP100	2	0	3	0	2	0	3	0	6	0
CENPX	2	0	1	0	< 1	0	3	0	3	0

Gallbladder (n = 379) topped with 10% ATM and followed by 7% BRCA2/FANCD1, rest 0%−3%, with driver mutations ATM 20, BRCA2 14, the rest 0–4.

Cervix cancer (n = 784) topped with 9% ATR and followed by 6% ATM, then by BLM, BRCA2/FANCD1, FANCI 4%, the rest 1%−3% With 10 driver mutations in BRCA2, 6 driver mutations in ATM and FANCD2, and 0–4 in the rest.

Eye cancer (n = 285) topped with 7% FAAP 20 and the rest 0%−3%, without a distinct number of driver mutations 0–2.

Bone cancer (n = 512) topped with 2% of ATM and FANCA without a distinct higher number of driver mutations.

Pediatric cancer (n = 5,749) without distinct mutation rate in 31 genes < 0%−1%, but with the highest number of driver mutations in FANCD2 = 36, the rest < 5, but an exception of ATM 16. The bold values indicate gene with high mutation rate.

### Tissue-type specificity is associated with the mutational signature of DDR/FA signaling-relevant genes across human cancers

Rigorous studies of DDR/FA signaling show that malfunctioning DDR/FA signaling in human cancer is multifaceted and influences cancer etiology, treatment sensitivity or resistance, and precision medicine. However, how the mutational signature of these FA signaling-relevant genes interacts with each type of human cancer has seldom been investigated.

Understanding these interactions is fundamental for facilitating cancer research and improving patient outcomes, given the multifaceted roles that FDDR/FA signaling plays in human cancer (2).

We studied the mutational status of 31 FA signaling-related genes in 15 different types of human cancers, including breast cancer (*n* = 11,657), brain cancer (*n* = 8,205), cervix cancer (*n* = 784), esophageal/stomach cancer (*n* = 4,994), eye cancer (*n* = 285), kidney cancer (*n* = 3,611), liver cancer (*n* = 1,829), lung cancer (*n* = 14,261), prostate cancer (*n* = 10,998), gallbladder cancer (*n* = 379), bladder cancer (*n* = 5,276), bone cancer (*n* = 512), bowel cancer (*n* = 7,661), skin cancer (*n* = 3,279), and pediatric cancer (*n* = 6,794).

As shown in Table 1, the gene with the highest mutated rate varied across cancer types, with each cancer type also featuring different driver genes and numbers of driver mutations in individual genes.

For example, ATM and BRCA2/FANCD1 had the highest mutated rate in only 5 out of 15 and 4 out of 15 cancer types, respectively. In contrast, FANCT had the highest mutation rate in breast and liver cancers, rather than BRCA or other prominent genes. Additionally, other genes such as ATR, RAD18, and FANCD2 were the most frequently mutated in specific cancer types such as skin, kidney, and eye cancers, respectively. These findings challenge the previous assumption that ATM and BRCA2 are universally high in all cancer types and suggest that therapeutic strategies targeting these genes should not be considered the primary option across all cancers. Each type of human cancer possesses its own unique genotype, which should be further investigated to improve cancer patient care.

### The mutational rate of each gene or the altered frequency of DDR/FA signaling is cancer-specific

Tissue-specific mutations are often used in the diagnosis and subtyping of human cancers. For example, certain mutations, such as HER2 amplification in breast cancer (25–27) or KRAS mutations in colorectal cancer (28, 29), help classify tumors into distinct subtypes, guiding treatment decisions and predicting prognosis.

To better understand these unique mutational signatures, we further examined the mutational signature within subtypes of human cancers (brain, breast, lung, and prostate) with large numbers (*n* > 8,000), allowing for separable subtypes. We found that the altered frequency of each gene or the DDR/FA signaling pathway resulting from these gene alterations is cancer-type or subtype-specific.

**Figure 2:**
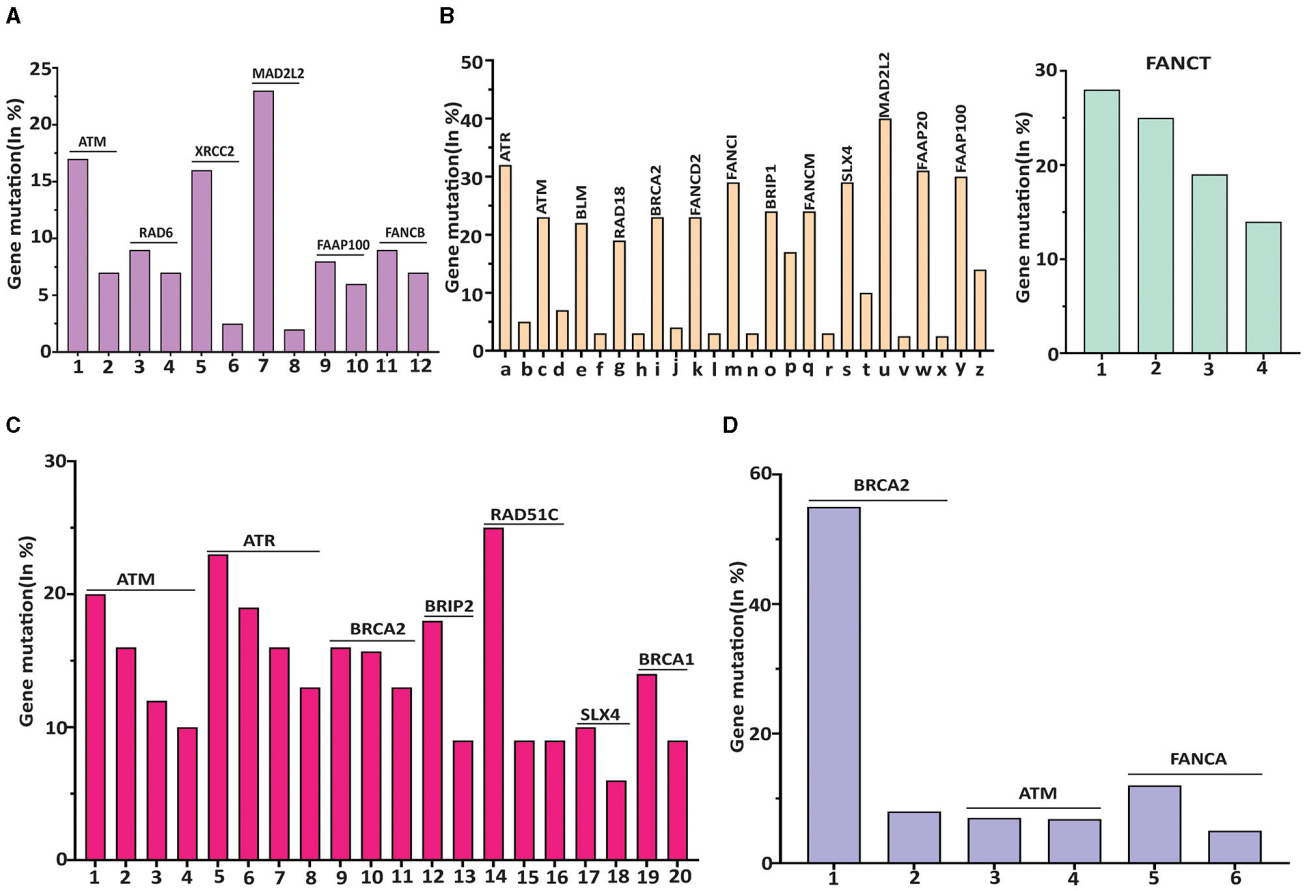
The highest mutated gene in each subtype of human cancer was featured with a different DDR/FA-relevant gene. (**A)** DDR/FA signaling-related genes vs. brain cancer. Mutated genes with >8% in the subtypes of brain cancer are ATM-16% in pediatric high-grade gliomas, Rad6-8% in rhabdoid tumor, XRCC2-16.5% in rhabdoid tumor, DAD2L2-21.5% in meningioma, FAAP100-8% in pediatric high-grade gliomas and FANCB-8% also in pediatric high-grade gliomas (the mutational rates of these genes in bars immediately following its top bar are also shown). The plotted subtypes of brain cancer are 1-pediatric high-grade glioma (PHGG), 2-glioma, 3-rhabdiod tumor (RT), 4-ependymoma, 5-RT, 6-PHGG, 7-Meningioma, 8-Paragangiloma, 9-PHGG, 10-oligoastrocytoma, 11-PHGG, and 12-ependymoma. **(B)** DDR/FA signaling-related genes vs. breast cancer. Mutated genes with greater than 20% in the subtypes of breast cancer are ATR-32%, ATM-24%, BLM-24%, RAD18-20%, BRCA2-23%, FANCD2-22%, FANCI-32%, BRIP1-25%, FANCM-23%, SLX-27%, 2MAD2L2/FANCV-37.5%, FAAP20-30%, FAAP100-28%, and FANCT-27% all in breast invasive cancer NOS (The mutational rates of these genes in bars immediately following its top bar are also shown). The plotted subtypes of breast cancer are (a) breast invasive cancer (BIC) NOS, (b) breast, (c) BIC NOS, (d) BIC, (e) BIC NOS, (f) breast invasive ductal carcinoma (BIDC), (g) BIC NOS, (h) breast invasive lobular carcinoma (BILC), (i) BIC NOS, (j) breast, (k) BIC NOS, (l) Breast, (m) BIC NOS, (n) BIDC, (o) BIC NOS, (p) adenoid cystic breast cancer (ACBC), (q) BIC NOC, (r) metaplastic breast cancer, (s) BIC NOS, (t) breast mixed ductal and lobular carcinoma (BMDLC), (u) BIC NOC, (v) BMDLC, (w) BIC NOS, (x) BMDLC, (y) BIC NOS, and (z) ACBC; and 1-BIC NOS, 2-BMDLC, 3-BILC, and 4-BIDC. **(C)** DDR/FA signaling related genes vs. lung cancer. Mutated genes with >10% in the subtypes of lung cancer are ATM in large cell lung carcinoma (LCLC, 18%), adenocarcinoma (16.5%), and large cell neuroendocrine carcinoma (LCNEC, 11.5%); ATR in combined small cell lung carcinoma (22.73%), adenocarcinoma (17%), squamous cell carcinoma (16.5%) and unknown primary origin (14%); BRCA2 in adenocarcinoma (16.5%), unknown primary origin (16%), and poorly differentiated non-small cell lung carcinoma (Pd-NSCLC,12.5%); BRIP1 in adenocarcinoma (18.5%); Rad51C in lung adenosquamous carcinoma (25%); SLX4 in unknown primary origin (11%); and BRCA1 in LCNEC (16.5%). The plotted subtypes of lung cancer are 1-large cell lung carcinoma (LCLC), 2-adenocarcinoma, 3-large cell neuroendocrine carcinoma (LCNEC), 4-non-small cell lung carcinoma (NSCLC), 5-combined SCLC, 6-adenocarcinoma, 7-squamous cell carcinoma, 8-unknown primary cancer (UPC), 9-adenocarcinoma, 10-UPC, 11-poorly differentiated NSCLC, 12-adenomcarcinoma, 13-LCLC, 14-lung adenosquamous carcinoma, 15-LCLC, 16-adenomcarcinoma, 17-UPC, 18-SCLC, 19-large cell endocrine carcinoma, and 20-LCLC. **(D)** DDR/FA signaling-related genes vs. prostate cancer. Mutated genes with >8% in the subtypes of prostate cancer are BRCA2 in prostate small cell carcinoma (54.5%) and prostate neuroendocrine carcinoma (9%), ATM in prostate neuroendocrine carcinoma (8%), and FANCA in prostate small cell carcinoma (12%). The plotted subtypes of prostate cancer are 1-prostate small cell carcinoma (PSCC), 2-prostate neuroendocrine carcinoma (PNEC), 3-PNEC, 4-prostate adenocarcinoma, 5-PSCC, and 6-PNEC.

As shown in Figure 2A, within subtypes of brain cancer, the top three mutation rates were MAD2L2/FANCV (21.49%) in meningioma, XRCC2/FANCU (17%) in rhabdoid tumors, and ATM (16%) in pediatric high-grade gliomas. Among breast cancer subtypes (Figure 2B), the top eight mutational rates were MAD2L2/FANCV (37.5%), FANCI (32%), ATR (32%), FAAP20 (30%), FAAP100 (28%), SLX4 (27%), FANCT (27%), and BRIP1 (26%) in breast invasive carcinoma NOS.

Even though FANCT did not have the highest mutational rate in breast invasive carcinoma NOS, it had the highest mutation rate in breast cancer overall (Table 1), likely due to its relatively higher mutational rates in breast mixed ductal and lobular carcinoma (25%), neoplasms (19%), and breast invasive ductal carcinoma (15.5%).

Regarding the subtypes of lung cancer (Figure 2C), the top five mutational rates are as follows: RAD51C/FANCO at 25% in lung adenosquamous carcinoma, ATR at 22.7% in combined small cell lung carcinoma, 17% in adenocarcinoma, and 16.5% in squamous cell carcinoma, BRIP1/FANCJ at 18.5% in adenocarcinoma, ATM at 18% in large cell lung carcinoma, and BRCA1/FANCS at 16.5% in large cell neuroendocrine carcinoma.

For prostate cancer subtypes (Figure 2D), the most frequently mutated genes are BRCA2/FANCD1 at 54.5% and FANCA at 12%, both in prostate small cell carcinoma. Other genes show mutation rates below 10%.

**Figure 3:**
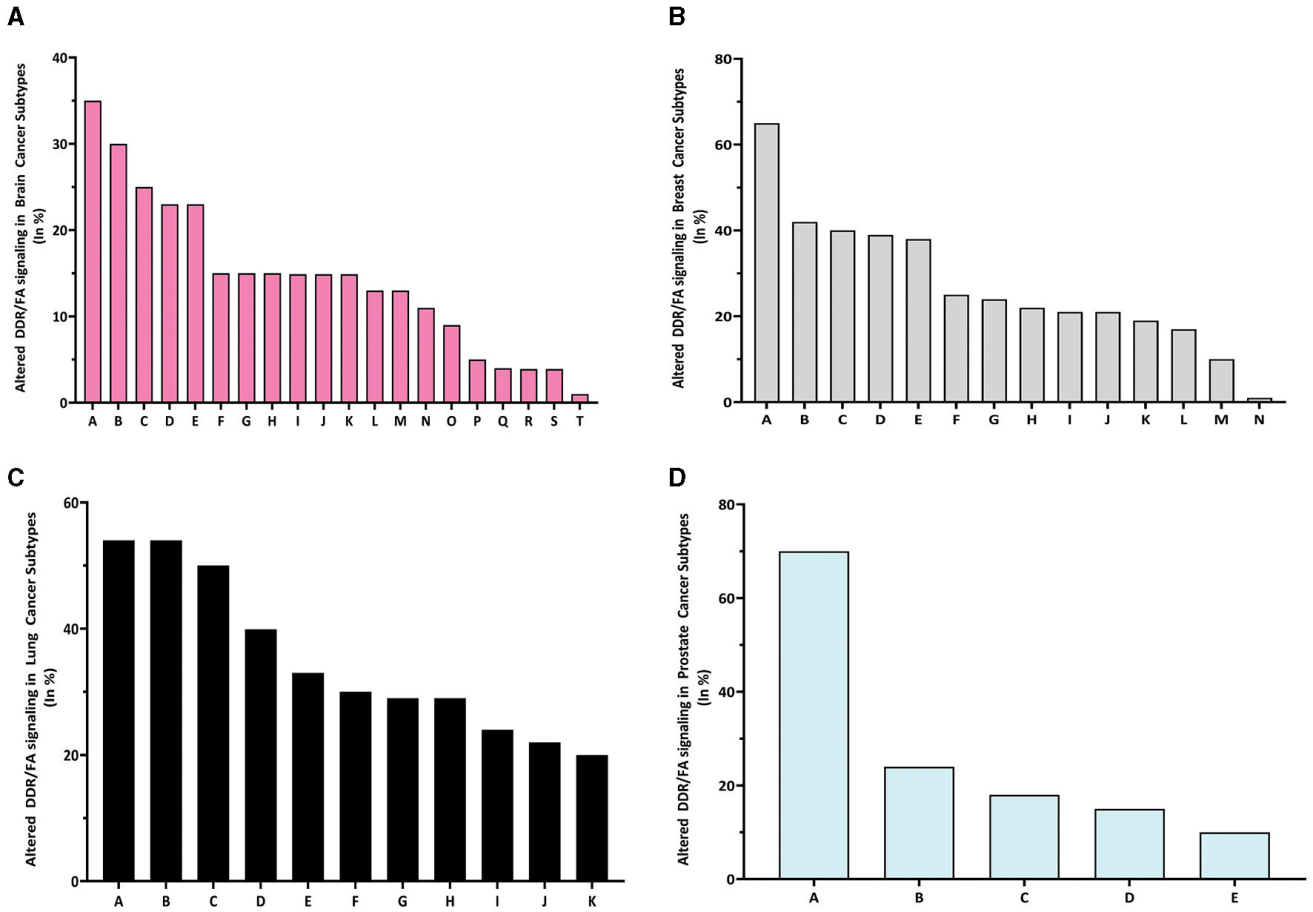
The altered rate of DDR/FA signaling resulting from 31 gene mutations in each type of human cancer is different. **(A)** The percentage of altered DDR/FA signaling in brain cancer is topped with pediatric high-grade gliomas (35%) among all listed subtypes (A-pediatric high-grade gliomas, B-glioblastoma, C-meningioma, D-astrocytoma, E-anaplastic oligodendroglioma, F-anaplastic astrocytoma, G-glioblastoma multiforme, H-atypical teratoid/rhabdoid tumor, I-anaplastic oligo astrocytoma, J-oligoastrocytoma oligodendroglioma, K-diffuse astrocytoma, L-diffuse glioma, M-pheochromocytoma, N-ependymomal tumor, O-paraganglioma, P-gliosarcoma, Q-glioma, R-medulloblastoma, S-pediatric low-grade gliomas, and T-pilocytic astrocytoma). **(B)** The percentage of altered DDR/FA signaling in breast cancer is topped with beast invasive cancer, NOS (65%) among all listed subtypes [A-beast invasive cancer, NOS, B-breast mixed ductal and lobular carcinoma, C-breast invasive carcinoma (NOS), D-breast invasive ductal carcinoma, E-invasive breast carcinoma, F-breast invasive lobular carcinoma, G-breast invasive carcinoma, NOS, H-breast invasive mixed mucinous carcinoma, I-adenoid cystic breast cancer, K-breast, L-metaplastic breast cancer, M-infiltrating ductal carcinoma, and N-benign phyllodes tumor of the breast]. **(C)** The percentage of altered DDR/FA signaling in lung cancer is topped with large cell lung carcinoma (55%) among all listed subtypes (A-large cell lung carcinoma, B-lung squamous cell carcinoma, C-adenocarcinoma, NOS, D-cancer of unknown primary, E-large cell neuroendocrine carcinoma, F-lung adenosquamous carcinoma, G-small cell lung cancer, H-lung Adenocarcinoma, I-poorly differentiated non-small cell lung cancer, J-non-small cell lung cancer, and K-combined small cell lung carcinoma). **(D)** The percentage of altered DDR/FA signaling in prostate cancer is topped with prostate small cell carcinoma (70%) among all listed subtypes (A-prostate small cell carcinoma, B-prostate adenocarcinoma, C-prostate, D-prostate neuroendocrine carcinoma and E-castration-resistant prostate cancer).

When examining the altered frequency of the whole DDR/FA signaling pathway resulting from these gene mutations across all cancer subtypes, the specific subtypes with the highest altered frequency are as follows: pediatric high-grade glioma (37%), breast invasive cancer (69%), large cell lung carcinoma (54.55%) and prostate small cell carcinoma (70.59%) in brain, breast, lung, and prostate cancers, respectively (Figures 3A–D). These results demonstrate that FA signaling and its related genes are frequently altered in human cancers in a manner that is both tissue-specific and cell-specific, which can guide treatment selection or precision medicine.

In summary, targeted therapies that focus on specific mutations or pathways are more effective in tumors harboring those mutations. This personalized approach will improve treatment outcomes and reduce unnecessary exposure to ineffective therapies.

### The association between altered FA signaling and patient outcomes varies across different types of human cancers

Mutations in specific genes can affect cancer cell behavior, such as growth rate, invasion, and response to therapy (1, 3, 30). Particularly, mutations in genes involved in DNA repair pathways, such as those involved in DDR/FA signaling, can lead to genomic instability, increase the risk of cancer development, and significantly affect responses to chemotherapy or targeted therapies. However, the relationship between altered FA signaling, defined by mutations in 31 related genes, and cancer patient outcomes has been scarcely examined.

By applying Logrank Tests (summarized in Table 2), we found the probability of overall survival was significantly higher in the unaltered FA signaling compared to the altered group in four out of 15 types of human cancers (breast, lung, prostate, bone; *p* < 0.0001).

**Table 2:** Probability of overall survival time, disease or progression-free period.

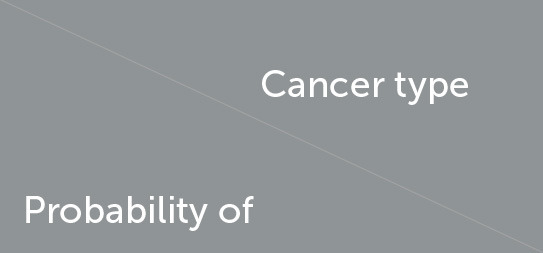	**Eye Pedia Eso/sto Cervix Gallbla. Brain**	**Skin**	**Bladder**	**Bowel**	**Prostate**	**Liver Kidney**	**Bone Breast**	**Lung**
Survival time higher in the Unaltered or					^***^		^***^	^***^
Altered group				^*^				
Disease-free higher in the Unaltered or						^*^		
Altered group			^***^					^*^
Progre. free higher in the Unaltered or					^*^			
Altered group		^*^		^***^				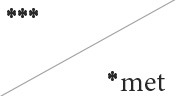

Blank-insignificant, ^*^ p < 0.05, and ^***^p < 0.0001; Group-the unaltered or altered FA signaling group.

In contrast, this difference was not significant in the other cancer types, with the exception of bowel cancer, where the probability was higher in the altered group than in the unaltered group (*p* < 0.05). In addition, the probability of being disease-free was significantly higher (*p* < 0.05) in the unaltered group for liver and kidney cancers, but the opposite was observed in bladder cancer (*p* < 0.001) and lung cancer (*p* < 0.05).

Regarding progression-free survival, the direction of probability varied: it was higher in the unaltered group for kidney cancer (*p* < 0.05) but higher in the altered group for skin cancer (*p* < 0.05) and bowel cancer (*p* < 0.001). Moreover, the probability of lung metastasis was significantly higher (*p* < 0.05) in the altered group. Since the brain is a primary site for lung metastasis, we also investigated central nervous system progression-free survival (CNS-PFS), which was significantly higher (*p* < 0.001) in the altered group.

**Figure 4:**
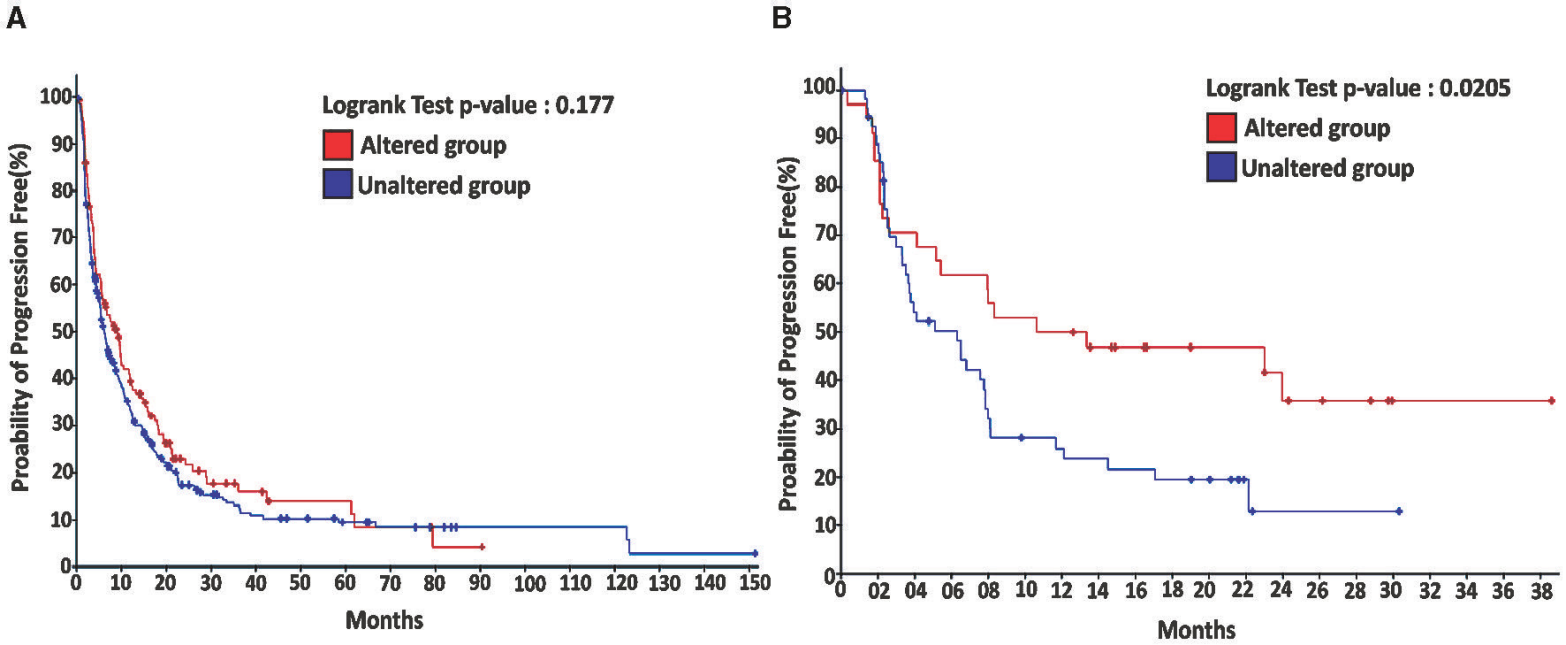
Immunotherapy promotes the probability of progression-free for patients with metastatic lung cancer harboring an altered DDR/FA signaling. **(A)** For metastatic lung cancer patients without immunotherapy, there is no significant difference between the patients carrying tumors with or without altered DDR/FA signaling. **(B)** For metastatic lung cancer patients treated with immunotherapy, patients carrying lung tumors harboring altered DDR/FA signaling have a significantly higher chance of progression-free than those carrying tumors without altered DDR/FA signaling, from the data shown within a comparable period of about 30 months. The probability of progression-free appears to be double that for the treated patients compared to the untreated group.

For non-small cell lung carcinoma, which often accompanies brain metastasis, we found that while the probability of progression-free survival was not significantly different (see Figure 4), it could be significantly improved (*p* < 0.05) when immunotherapy was applied. These results demonstrate that the impact of altered FA signaling on patient outcomes, whether positive or negative, generally depends on tumor type and the specific therapies applied, such as immunotherapy.

## Discussion

Dysfunction in DDR/FA signaling compromises genome integrity, making cells more susceptible to both endogenous and exogenous DNA-damaging agents (1, 3, 6). Disruptions in these pathways lead to genomic instability, which significantly increases the risk of tumorigenesis (31–34). Mutation in genes responsible for regulating DDR/FA signaling in various cancers and cancer subtypes predisposes individuals to various disorders (2, 35). While defective gene products in the DDR/FA signaling pathway are known to play a role in DNA repair, their functions are not yet fully understood (1, 35). Our study on the mutational patterns of DDR/FA signaling-relevant genes in human cancer not only fills gaps in the current research but also improves our understanding of carcinogenesis, offering new avenues for basic, translational, and clinical cancer research.

Multiple studies across various cohort samples have identified at least 13 FA genes responsible for high-risk breast cancer predisposition (36). To date, over 23 FA genes have been implicated in FA disorders (37).

In another study involving 1,021 hereditary cancer patients, 35 pathogenic variants were identified across eight genes, with FANCA emerging as the most frequently mutated FA gene in both breast cancer/breast and ovarian cancer cases (38). Interestingly, a study by Chang et al. (22) examined 10 different cohorts, comprising 3,235 AML and 1,024 MDS patients, totaling 4,259 patients with myelodysplastic syndromes or acute myeloid leukemia.

This study revealed the potential functional involvement of FA pathway gene expression and mutations in the clinical traits, particularly in patients with mutations in FANCA, FANCE, FANCL, FANCM, SLX4, and FANCD2 (22). These studies highlight the importance of DDR/FA signaling in human cancer development and progression, further motivating comprehensive research in this area.

Our DNA-seq analysis of 76,639 pan-cancer samples identified 31 DDR/FA signaling genes, with ataxia-telangiectasia mutated (ATM) showing the highest mutation rate at 5%, followed by BRCA2/FANCD1 at 4%. These findings are consistent with those from Moslemi et al., who also identified ATM as the most frequently mutated gene (20, 22, 39).

We also observed that ATM and BRCA2/FANCD1 consistently exhibit the highest mutation rates across different tissue-specific cancer types, a pattern corroborated by other mutational screening studies (22, 40, 41). Given the significant biological roles of these genes, various studies have explored the efficacy of ATM inhibitors in ATM-deficient lung, prostate, and pancreatic cancer cells (42–45).

Interestingly, our analysis of the 31-DDR/FA-gene mutation signature, derived from one of the largest sample sizes to date, supports the development of diagnostic techniques in conjunction with new approaches such as immunotherapy (46, 47).

Recent studies using murine models have demonstrated the potential of immunotherapy in treating human FA-AML (48, 49), highlighting the need for a comprehensive, novel FA mutation database that can aid in the advancement of immunotherapy for this rare autosomal disorder.

Expanding the sample size and broadening the scope of clinical prognosis and survival outcomes are essential for providing a more thorough and unbiased assessment of the relationship between FA expression, mutation, and the prognosis of FA-associated cancers. By incorporating a wider range of patients, researchers can gain deeper insights into the complexities of FA-related cancer development, progression, and treatment outcomes.

Moreover, enhancing the dissemination of information regarding FA status and prognostic indicators to patients is essential for early detection, personalized treatment approaches, and improved outcomes (1–3, 35). This effort could include implementing educational initiatives, genetic counseling programs, and screening protocols to ensure that individuals with FA receive timely and appropriate medical care. Addressing these challenges with a more inclusive approach to research and patient care can significantly advance our understanding of FA-associated cancers and improve clinical management strategies for affected individuals.

In particular, our study of somatic mutation can help establish correlations between FA gene expression/mutations and the survival prognosis of cancer patients, which would lead to the development of predictive tools, such as Nomograms, to estimate overall survival rates based on FA-related factors and the creation of efficient FA-related random forest/decision tree classifiers to assess the cytogenetic risk of cancer patients. By examining the genetic and expression profiles of DDR/FA signaling genes, the study aimed to elucidate their role in cancer development, progression, and patient outcomes. These predictive models would be valuable tools for clinicians, guiding treatment decisions and improving prognosis assessments.

Despite the benefits of studying somatic gene mutations in human cancer, much remains unknown about how these mutations function during tumor development and progression. Although we have identified potential driving mutations, it is still unclear whether they truly drive tumor development and progression. Among these, FANCT stands out, as its mutation rate is higher than all others in breast tumorigenesis. This gene warrants immediate attention for further basic, translational, and clinical research to advance our knowledge and ultimately improve patient care.

## Materials and methods

### Publicly available DNA sequence datasets

This study utilized publicly available DNA sequence datasets to analyze the mutation rates of 31 genes involved in DDR/FA signaling (including ATM, ATR, BLM, HRR6, RAD18, FANCA, FANCB, FANCC, BRCA2, FANCD2, FANCE, FANCF, FANCG, FANCI, BRIP1, FANCL, FANCM, PALB2, RAD51C, SLX4, ERCC4, RAD51, BRCA1, UBE2T, XRCC2, MAD2L2, RFWD3, FAAP20, FAAP24, FAAP100, and CENPX). These analyses were conducted using the c-BioPortal platform (24, 50), which facilitated the determination of mutation rates and the frequency of altered DDR/FA signaling across a variety of human cancers.

To perform the analysis, we entered the symbols of the 31 genes into the c-BioPortal system and selected options to automatically compute mutual exclusivity and/or co-occurrence among the genes. Additionally, the platform allowed us to perform cross-cancer queries or focus on specific cancer types.

The DNA sequence datasets were sourced from various studies and repositories, including TCGA, MSKCC, BGI, BCCRC, Nature, Nature Genetics, Cell, Science, AMC, Cancer Cell, PNAS, and others. For pan-cancer samples, the datasets were originally derived from studies such as MSK Nat Med 2017 (*n* = 10,945), Nat Genet 2019 (*n* = 1,661), Clin Cancer Res 2020 (*n* = 106), Nat Genet 2020 (*n* = 24,246), Cell 2021 (*n* = 25,775), UMich Nature 2017 (*n* = 500), Broad/Dana-Farber Nat Genet 2018 (*n* = 249), Multi-Institute Nature 2018 (*n* = 141), ICGC/TCGA Nature 2020 (*n* = 2,922), and OrigiNed Nature 2022 (*n* = 10,194), although not all samples were recorded with usable DNA sequences.

For specific cancer types, the datasets were as follows:

**Breast Cancer** (*n* = 11,657): 26 studies from journals including Cell, Cancer Cell, Nature, Cancer Discovery, MSK, Nat Genet, and others, including TCGA.
**Lung Cancer** (*n* = 14,261): 34 studies published in various journals or reported to TCGA, etc.
**Bladder Cancer** (*n* = 5,276): 21 studies.
**Brain Cancer** (*n* = 8,205): 26 studies.
**Kidney Cancer** (*n* = 3,611): 17 studies.
**Prostate Cancer** (*n* = 10,996): 13 studies.
**Esophageal/Stomach Cancer** (*n* = 4,994): 20 studies.
**Bowel Cancer** (*n* = 7,661): 21 studies.
**Liver Cancer** (*n* = 1,829): 12 studies.
**Skin Cancer** (*n* = 3,279): 19 studies.
**Gallbladder Cancer** (*n* = 379): 3 studies.
**Cervix Cancer** (*n* = 784): 3 studies.
**Eye Cancer** (*n* = 285): 5 studies.
**Bone Cancer** (*n* = 512): 3 studies.
**Pediatric Cancer** (*n* = 5,749): 14 studies.

All DNA sequence datasets were publicly accessible through c-BioPortal and were used for mutational analysis, focusing only on those cancer samples that produced high-quality DNA sequences.

### Statistical analysis

The correlations between altered DDR/FA signaling and cancer prognosis were analyzed using the Chi-square test. A *p*-value of < 0.05 was considered statistically significant.

## Data Availability

The datasets presented in this study can be found in online repositories. The names of the repository/repositories and accession number(s) can be found in the article.
